# Psychological Screening and Tracking of Athletes and Digital Mental Health Solutions in a Hybrid Model of Care: Mini Review

**DOI:** 10.2196/22755

**Published:** 2020-12-14

**Authors:** Luke Balcombe, Diego De Leo

**Affiliations:** 1 School of Health and Sport Science University of the Sunshine Coast Sunshine Coast Australia; 2 Australian Institute for Suicide Research and Prevention Griffith University Brisbane Australia

**Keywords:** athletes, screening, tracking, engagement, well-being, stress, adjustment, COVID-19, hybrid model of care, digital mental health, machine learning, artificial intelligence

## Abstract

**Background:**

There is a persistent need for mental ill-health prevention and intervention among *at-risk* and vulnerable subpopulations. Major disruptions to life, such as the COVID-19 pandemic, present an opportunity for a better understanding of the experience of stressors and vulnerability. Faster and better ways of psychological screening and tracking are more generally required in response to the increased demand upon mental health care services. The argument that mental and physical health should be considered together as part of a biopsychosocial approach is garnering acceptance in elite athlete literature. However, the sporting population are unique in that there is an existing stigma of mental health, an underrecognition of mental ill-health, and engagement difficulties that have hindered research, prevention, and intervention efforts.

**Objective:**

The aims of this paper are to summarize and evaluate the literature on athletes’ increased vulnerability to mental ill-health and digital mental health solutions as a complement to prevention and intervention, and to show relationships between athlete mental health problems and resilience as well as digital mental health screening and tracking, and faster and better treatment algorithms.

**Methods:**

This mini review shapes literature in the fields of athlete mental health and digital mental health by summarizing and evaluating journal and review articles drawn from PubMed Central and the Directory of Open Access Journals.

**Results:**

Consensus statements and systematic reviews indicated that elite athletes have comparable rates of mental ill-health prevalence to the general population. However, peculiar subgroups require disentangling. Innovative expansion of data collection and analytics is required to respond to engagement issues and advance research and treatment programs in the process. Digital platforms, machine learning, deep learning, and artificial intelligence are useful for mental health screening and tracking in various subpopulations. It is necessary to determine appropriate conditions for algorithms for use in recommendations. Partnered with real-time automation and machine learning models, valid and reliable behavior sensing, digital mental health screening, and tracking tools have the potential to drive a consolidated, measurable, and balanced risk assessment and management strategy for the prevention and intervention of the sequelae of mental ill-health.

**Conclusions:**

Athletes are an *at-risk* subpopulation for mental health problems. However, a subgroup of high-level athletes displayed a resilience that helped them to positively adjust after a period of *overwhelming* stress. Further consideration of stress and adjustments in brief screening tools is recommended to validate this finding. There is an unrealized potential for broadening the scope of mental health, especially symptom and disorder interpretation. Digital platforms for psychological screening and tracking have been widely used among general populations, but there is yet to be an eminent athlete version. Sports in combination with mental health education should address the barriers to help-seeking by increasing awareness, from mental ill-health to positive functioning. A hybrid model of care is recommended, combining traditional face-to-face approaches along with innovative and evaluated digital technologies, that may be used in prevention and early intervention strategies.

## Introduction

### Elite Athlete Mental Health Phenomenon and Digital Mental Health Potential

Elite athlete mental health problems recently attracted great discussion as a worldwide relevant phenomenon [1], including reference to sporting factors, such as injury, overtraining, burnout, and career termination as well as nonsporting factors [2,3]. The array of athlete-specific mental health symptoms and disorders such as distress, depression and suicide, anxiety and stress, overtraining, sport-related concussion, substance misuse, sleeping and eating disorders, posttraumatic stress disorder (PTSD) and other trauma-related disorders, bipolar and psychotic disorders, attention-deficit/hyperactivity disorder, gambling disorder, and other behavioral addictions [4-6], are complicated by a range of social and psychological factors as well as attributes including personality, sexuality and gender issues, hazing, bullying, sexual misconduct, and transition from sport [6].

An increased interest in understanding the complexity of mental health among elite athletes has led to the empirical development [7] and validation [8] of athlete-specific questionnaires that are acceptable and appropriate for measuring mental health symptoms in the athlete environment. There remains an unrealized potential for universal electronic screening for mental health concerns, especially as a preparticipation examination when competitive athletes transition to a higher level (administered by sporting organizations) [9]. The early detection of mental health disorders has been proposed as part of an athlete-specific early intervention framework involving web-based consultations [10]. There is potential for this approach to be expanded at times of life changes, transition, and retirement. However, flexibility and pragmatism are required to apply digital mental health tools such as smartphone apps cognizant of the dichotomous directions and efforts that divide this space and limit its potential (randomized trials vs pragmatic studies, precision medicine vs population health, free market vs regulation, consumer vs clinical uses, big data vs privacy, and open vs proprietary software) [11].

### Athlete Mental Health Challenges Amid COVID-19

Although preceded by many other epidemics caused by infectious disease, the COVID-19 pandemic is worthy of particular exploration because of a trend of increasing emergence of novel pathogens from nonhuman hosts. Major worldwide disruptions to habits and customs have impacted elite athletes’ training and competition. Uncharted challenges have been posed for those engaged in sport, with preliminary issues associated with social isolation, career disruption, qualification process uncertainty, and unconventional and limited access to effective training environments and partners [12]. This has been compounded by uncertainty around sporting events, which led to a negative response over a period of weeks to months [13]. However, some positives were drawn by the adaptive and resourceful group of those with well-formed athletic identities: a chance to refresh, time for injury recovery, and time for honing practice to fill perceived gaps in development [12]. Examples of novel training methods were shared by some high-profile elite athletes via social media during the COVID-19 lockdown.

There are calls to better understand the disruption and psychosocial consequences arising from the COVID-19 pandemic and predicted mental health case surge across general populations [14,15]. The urgent mental health care response makes it worthwhile to consider opportunities to pivot from already ongoing studies [16]. Research that disentangles aspects of athletes’ experience of stress and adjustment issues during changes and transitions can provide important contributions to early intervention frameworks or models of care as well as educational resources. A clearer picture of physical and mental activity behaviors, well-being, stressors, and adjustment is required from athlete-specific screening and monitoring to better understand the holistic health of high-level athletes [17,18].

Recent longitudinal research with high-level athletes (Simons et al, unpublished data, 2020) and college students [19] identified these subgroups as *at risk* of mental health problems amid COVID-19. These studies included online (smartphone compatible) assessments. The results suggested the influence of *overwhelming* stress and changes in mental health and behaviors related to the pandemic situation. In college students, this was negatively altered with a restrictive effect related to the pandemic proximity, media coverage, and policy. The mixed linear model of smartphone mobile sensing and self-reported mental health questions [19] was applied in this study. In synthesis, the main theoretical basis informing these studies is the hybrid model of care that identifies patients receiving both in-person and online interventions for diagnosis, therapy, and monitoring [10,20-22].

### Digital Mental Health Opportunity Amid COVID-19

Digital mental health interventions have been proposed as a flexible and cost-effective way to reduce stigma and the treatment gap among university students [23]. However, the limitations are low adherence rates and questionable long-term efficacy in real-world settings. A collaborative effort to confront digital and system challenges within the general population will be required to “realize the vital goals of mental health prevention and support by providing a means to measure and track population mental health” [24]. A road map, a communication tool that articulates the strategic thinking of the goal and plan, is required to strengthen global mental health systems to tackle the impact of the COVID-19 pandemic [25]. Maulik et al [25] drew upon key sources and accumulated knowledge of mental health systems worldwide to provide a perspective on practical steps for implementation.

An integrated blueprint to deliver digital mental health screening and tracking highlighted the need to provide specifications for vulnerable subpopulations such as children, college and university students, domestic violence victims, frontline heath care workers, low socioeconomic groups, those with mental health disorders, and older adults [26]. Athletes were initially included with this group. However, it should be clarified that high-level athletes are a peculiar *at-risk* group because there is an increased vulnerability yet resilience that requires further investigation and articulation. There is an opportunity to enable digital tools and artificial intelligence in athlete mental health screening and tracking to collect real-time data in a hybrid model of care (combining online and offline data with in-person data) to enrich research data to support better models of care [26]. This leads to the research question: should digital screening and tracking tools be tailored toward adjustment and stress in *at-risk* subpopulations such as athletes to assist the management of their mental health?

## Methods

This mini review shapes literature in the fields of athlete mental health and digital mental health by summarizing and evaluating journal and review articles drawn from PubMed Central and the Directory of Open Access Journals (generally from within the last 4 years). There are also internet sources (refereed electronic journals), a thesis, conference proceedings, books and websites to complement the primary research. A story has been developed to show relationships between athlete mental health problems and resilience as well as digital mental health innovation with recommendations presented for research prospects in the near future.

## Results

### Elite Athlete Mental Health Screening and Tracking

The use of technology in elite athlete mental health screening has been limited to internet-based surveys. A brief, fully automated internet-based pilot study of mental health help-seeking interventions [27] addressed mental health literacy and found that stigma can lead to underreporting and inhibit help-seeking behavior. This led to a model of care and an inference that young elite athletes are particularly unlikely to seek help compared to nonathletes. A self-report internet-based prevalence study [28] established that just under half of the surveyed Australian high-level athletes met the criteria for at least one mental health problem. However, there were engagement issues with insufficient power in the analyses to draw firm conclusions.

A qualitative study transformed from a psychological protective and risk factors screening (an online pilot study) in response to engagement difficulties [29]. An investigation of psychological distress in current and retired Australian elite athletes resulted in novel findings on how accumulative stress manifests into more intense and severe symptoms over time. The vortex of upward and downward spirals was described as the spiraling of states between positive and negative outcomes, a turbulent transitional period where retired elite athletes reported it took 1-3 years for the balance to be restored in their mental health. This highlights the importance of a tailored prevention and early intervention strategy that effectively engages athletes in the screening of psychological protective and risk factors, and tracking that seeks to pinpoint an understanding of how these factors are associated with psychological symptoms, disorders, and abnormal behavior.

Short-form mental health screening instruments were applied with substantial variability in American collegiate athletes, which led to a call for brief validated *electronic* survey instruments that can be interpreted by nonspecialists and have a utility of screening for symptoms or risk factors of specific mental health disorders [9]. An example of such is the Athlete Psychological Strain Questionnaire (APSQ), a brief self-report screening tool by Rice et al [30]. The APSQ was implemented with male elite professional athletes from three national Australian sporting codes (Australian football, cricket, and soccer; n=1007, 78.6% participation rate). Twelve items were developed in the three thematic areas of self-regulation, performance, and external coping to assess for psychological distress and well-being, difficulties with team-based interactions, impaired impulse control and frustration tolerance, worries related to athletic performance and training stress, and transition to life beyond professional athletic pursuits. However, the sensitivity and specificity of the APSQ was designed to identify the behaviors that suggested underlying distress. Modeled on the widely used and validated Kessler 10 and with validity and reliability established from psychometric evaluations [30], the APSQ includes a range of cut-off points reflecting moderate, high, and very high scores, and is the recommended triage screening tool within the International Olympic Committee’s Sports Mental Health Assessment Tool [8].

The lack of robust empirical data on mental health symptoms in currently competing athletes [31] led to a cross-sectional, anonymous, online survey of prevalence and correlates of mental health symptoms in high-level athletes (n=810) at the Australian Institute of Sport (AIS). There was a good participation rate (51.7%) with a significantly higher report of *high to very high* psychological distress in high-level athletes compared to general community norms (17.7% vs 9.5%, respectively). These significantly high levels of distress (17.7%) correspond with findings of 16% of meta-analyses from 34 original studies, with 1 at low risk of bias [4]. The extent of other mental health symptoms and disorders ranged from 19% for alcohol misuse to 34% for anxiety and depression for current elite athletes and to 26% for anxiety and depression for former elite athletes. Valid comparisons with the general population were not established by these authors. However, it was surmised that these levels may be slightly higher than in the general population, leading to the call for further research that focuses on the development of reliable and valid screening instruments.

Knowledge of how the mental ill-health sequelae manifests in elite athletes is not yet established in the literature [30], which in turn limits knowledge of impeding factors. The impact of the COVID-19 lockdown upon the mental well-being of high-level athletes was captured in a longitudinal cohort study by Simons et al (unpublished data, 2020), with research support and a sport psychology network to assist athletes [32] from the Queensland Academy of Sport (Australia). A monitoring study (n=15) ensued after online screening of stressors with a smartphone-compatible questionnaire (face-to-face was nullified). Relocation and self-quarantine (coinciding with the implementation of the harshest restrictions of COVID-19) were related to a high risk of adjustment disorder. Mental well-being was negatively impacted during the lockdown period with reports of being *overwhelmed* beyond their sporting careers (into their holistic existence). However, the return to baseline well-being during this period indicated resilience and successful implementation of coping strategies and (self) interventions.

### Elite Athlete Mental Health Literature Reviews and Consensus Statements

The generally shared viewpoint of meta-analytic reviews is that elite athletes experience a broadly comparable risk of high-prevalence mental disorders (eg, anxiety and depression) relative to the general population [2]. More specifically, there are comparable rates of depression [4,30,33] as well as anxiety, posttraumatic stress, and sleep disorder [4,30]. Athletes are unique in that there is an existing stigma of mental health and underrecognition of mental illness as well as the possibility of simultaneously having both positive mental health and experiences of mental illness [34]. The range of both athlete-specific and general risk factors associated with mental ill-health in elite athletes led to a comprehensive early intervention framework [10]. A hybrid model of care (face-to-face or by telephone or web-enabled consultations) was used to target the limiting factors of athletes’ psychological processes and intervention via the mental health referral network at the AIS. The intent of the AIS early intervention model of care is for the referred practitioner to work one-on-one with the athlete to address their needs in a restoration approach to mental health and functioning [35].

Elite athlete mental health research has evolved as a subset of psychiatry and psychology. Significant recent contributions from sports psychiatry and sport psychology (including clinical psychology) resulted in consensus, expert, and position statements on the individual, cultural, and environmental factors that affect the well-being of the athlete and inform the prevention and treatment of mental health symptoms and disorders [1] (see [3,5,36-40]). A key recommendation is the need for action toward greater consistency in the development, evaluation, and reporting of mental health awareness programs [36]. It was noted that there is “no evidence or consensus-based guidelines for diagnosis and management of mental health symptoms and disorders in elite athletes” [5]. A previous systematic review [41] concluded that a cautious approach is required in determining effective evidence and theory-based intervention programs with unbiased, high-quality methods. Breslin et al [41] outlined blind randomized longitudinal studies as being needed with larger sample sizes and outcomes measured with validated measurement tools that account for behavior change related to mental health literacy. There has been a lack of evidence and discussion of how technology may be applied in the development and evaluation of research or mental health awareness, prevention, and intervention programs.

There are calls for a better understanding of sport as a subculture within society [5,37,42] and for future studies to highlight positive mental health outcomes within athlete subgroups. Investigations into the athletic identity, individual and subgroup character, and cultural traits led to further issues being identified with regard to help-seeking barriers [42]. In particular, Castaldelli-Maia et al [42] found that elite sport culture includes some features that increase the likelihood of athletes with mental health symptoms and disorders. A summary of barriers to athletes seeking mental health treatment was presented including stigma, low mental health literacy, negative past experiences with mental health treatment seeking, busy schedules, and hypermasculinity. Male high-performance athletes were previously reported as 52% less likely than their female counterparts to report mild or more severe depressive symptoms [34]. The proposed link between disclosure of mental health symptoms and disorders, and the stigma of being perceived as *weak* [42] is central to the suggestion that brief antistigma interventions offer promise as an initial step to overcome help-seeking barriers. Further research is required for more focused and tailored interventions for subgroups. For example, it was advised to expand upon gender differences (larger, representative female athlete samples are required) [8].

Acknowledgement of the global challenge of increasing mental health literacy of elite athletes and reducing help-seeking barriers prompted the call for management strategies to address all contributors to mental health symptoms and consider biopsychosocial factors relevant to athletes [5]. Rice et al [30] noted the existence of athlete-centric models of care [3,43]. However, an early intervention framework or model of care is required to support and respond to the mental health needs of this group [10]. The design, implementation, and evaluation of intervention programs may be derived from appropriate theories and models [36]. This implies the merits of reverse engineering. It is not yet established how technology may be applied by functional specialists for the delivery of specific mental health prevention and care outcomes. This gap in the literature highlights the importance of developing and using professionals who can bridge the gap between data science, technology, and organizational strategy.

A key finding for engagement between sporting organizations, elite athletes, and mental health care services highlighted the need to “screen for psychological distress in athletes who may otherwise appear to be well-functioning, to provide timely, optimal treatment” [31]. This is an important consideration as national funding agencies, Olympic committees, federal governments, and sport organizations roll out strategies to combat mental ill-health during COVID-19 [12]. Schinke et al [12] suggested to learn from lessons gained through “autonomy, ingenuity, resilience, life balance, mindfulness” among other skill sets. There is a suggestion to consider cases of *false negatives* and *false positives*, and to capture such distinctions in the screening of the range of psychological function, from positive attributes to severe distress. However, elite athlete mental health prevention and early intervention frameworks have yet to expand beyond a web-based consultative approach to incorporate the potential of digital mental health, especially with regard to the use of technology and human-computer interactions (HCIs).

A theoretical framework needs to recognize the impact of general and athlete-specific risk factors, engage key individuals that may identify and promote athlete mental health, and be adaptable and responsive to varied career stages and mental health states [10]. There is a wealth of theoretical knowledge and insight in the recent literature, yet there is a need for summary, evaluation, and recommendations on ways to effectively engage athletes via technology and make well-designed use of models of care, and adapt or extend them to increase their explanatory power. Furthermore, there is a welcome emphasis on commitment to holistic health and a prevention and early intervention framework. These developments help to redress the balance in favor of mental and physical health being considered together with support for biopsychosocial studies of mental health. It offers a balance with regard to the elite sporting culture, and there is clearly global interest in the results and implications.

## Discussion

### COVID-19 Pandemic Impact on Mental Health a Catalyst for Change in Screening

There is a need to transform key sources and worldwide accumulated knowledge of mental health systems into insightful findings that can be effectively grasped. The limitations of organizing data scientists and difficulties in upskilling mental health researchers in data science (database systems, programming and data analytics, data mining and management, machine learning, and visual analytics) has hindered the practical steps needed to strengthen mental health systems. The quality of collaboration in elite athlete mental health consensus statements, systematic reviews, and models of care suggests that this field is suitable for the testing of digital mental health. Inroads made thus far, with some sporting organizations having opened up their doors to research via a biopsychosocial approach, lead to the proposal that persistence, data science, and digital mental health methods will result in articulation of how the mental ill-health sequelae manifests in athletes.

A lack of availability and accessibility of acceptable resources and use of counselling during the COVID-19 pandemic [44] points toward evidence of the void that a hybrid model of care (telehealth, digital mental health, and face-to-face) has a great opportunity to contribute to. There is a high potential for data-driven innovation strategies to expand knowledge of how the mental ill-health sequelae manifests in athletes. Machine learning in particular may lead to change more generally if more precise ethics are implemented, resulting in better and faster screening, assessment, and management.

The COVID-19 pandemic has had a profound psychological and social effect [45] with severe psychiatric impacts on the community [46] and increased suicide rates expected [47], especially linked to higher unemployment [48]. Research studies are needed on how mental health consequences can be mitigated during and after the COVID-19 pandemic with a focus on decreasing stress, anxiety, fears, and loneliness in the general population [45]. Sher [45] pressed for urgency but predicted suicidal behavior is likely to be present for a long time and peak later than the actual pandemic. As a result of this catalyst for change, it was suggested to focus on vulnerable subpopulations. Patients at risk for suicide require psychiatric services and monitoring to ensure safety [49]. Schrieber and Culpepper [49] explained that a holistic understanding of the clinical features and course of mental ill-health in subpopulations are prerequisites for the screening, assessment, and management (including prevention and intervention) of people and patients who are vulnerable to develop a clearer picture of the “underlying factors of psychiatric disorders, precipitating events, and ongoing life circumstances” with recommended remedy from “medications, counselling, and involvement of friends, family, and religious/community groups as appropriate.”

There is preliminary evidence among general populations that the COVID-19 pandemic may be associated with psychiatric symptoms that do not necessarily rise to the level of a psychiatric disorder as well as full-blown anxiety disorders, depressive disorders, insomnia disorder, and PTSD [50]. Stein [50] found the pathogenesis of psychiatric symptoms and disorders may include biologic and psychosocial factors, which were noted as possibly increasing the risk of suicidal ideation and behavior because of the array of hardships imposed by the pandemic. This review was especially concerned for patients with COVID-19 and clinicians who treat patients as well as individuals in quarantine. It expresses the overwhelming need to simultaneously screen, track, and treat the “new onset or exacerbation of subsyndromal psychiatric symptoms as well as full-blown psychiatric disorders, including anxiety disorders, depressive disorders, PTSD, or substance use disorders.”

Answering the call for a generalized prevention and intervention tool [50], a first-line screening tool called the Mental Health Quotient (MHQ) was developed to provide for the individual perspective (a profile of mental health challenges and positive well-being) [24]. It screens for concerns and abilities across a results profile while developing a complete picture and unbiased insights of reported symptoms and functions. It is an efficient and user-friendly online assessment of population mental health and well-being. It is suitable for use in primary care and psychiatric clinics to identify *at-risk* individuals and subgroups. It provides information for diagnosis across 10 disorders after a comprehensive review of symptoms across 126 commonly used psychiatric assessment tools. A distinguishing feature is that it is not constrained by the clinical classification systems, the Diagnostic and Statistical Manual of Mental Disorders (DSM) [51] or the International Classification of Diseases (ICD) [52]. The MHQ is useful in identifying people who do not qualify for a particular disorder yet need help because of a large number of severe clinical symptoms (from an anonymous screening that provides a score and full individual report that encourages honest self-reporting). Validity was suggested from demonstration of a close alignment between MHQ scores and the degree to which people meet DSM-5 diagnostic criteria as well as alignment with known epidemiological estimates along with various dimensions including annual prevalence rates of mental health disorders and age and gender difference findings [24]. However, the MHQ is not known to have been tested by others for its validity.

The MHQ is generally useful to target subsyndromal symptoms before it becomes distress or impairment [24]. There is expected to be a greater demand for evidence-based mental health interventions as cultural change prompts more positive perceptions of help-seeking [35]. The athletic culture and dichotomous mental health states and associated biopsychosocial investigations provide a context to compare screening methods and results. The evaluation of the APSQ screening tool [30] was a timely development especially with regard to its validity and reliability [8]. It is a triage screening tool that is relevant worldwide, feeds into an early intervention framework [10], and is connected with the established national Mental Health Referral Network, which presents opportunities for expansion and data capture, informing future education-based initiatives and aims at positively affecting the broader culture around mental health and help-seeking behaviors [35].

A synthesis of a select range of psychological issues and mental health disorders in competitive athletes was intended to assist team physicians and other members of the athletic care network with detection, treatment, and prevention [6]. Screening with Qualtrics (brief psychological survey accessible on a digital device from anywhere) clinically assessed the coping and psychological functioning of nearly 6000 student athletes during the COVID-19 pandemic [44]. Petrie [44] noted small but sizable levels of severe impairment in terms of depressive symptoms, psychological distress, and dissatisfaction with life but a higher incidence of moderate (or subclinical) levels on these measures. Findings between elite [42] and student athletes [44] indicate that these subgroups of athletes face significant challenges with ongoing mental health stigma. Cross-sectional results of high-level (Simons et al, unpublished data, 2020) and student athlete [44] studies suggest a prevalence of depression and distress during the COVID-19 pandemic. However, the study with high-level athletes (Simons et al, unpublished data, 2020) demonstrated a return to baseline levels during the lockdown period, suggesting a possible differentiation for the positive (limited by a small number of participants in the monitoring study).

### Broadening the Scope of Elite Athlete Mental Health Symptom and Disorder Interpretation

There have been concerns raised for more than a decade about misdiagnosis arising from current symptom-based DSM and ICD diagnostic criteria for mental disorders, which “are prone to yielding false positives because they ignore the context of symptoms” [53]. However, *false positives* have been widely argued to not be a benign flaw because support and treatment for “disorders” are beneficial to those experiencing emotional problems. Harmful dysfunction analysis has been described for “disordered” classification if human beings fail to satisfy the new social demands [53].

There are *false negative* cases that may arise in elite athletes from dishonest screening responses out of fear of selection or losing a position and, thus, presenting difficulties for detection by functional specialists. There are also different interpretations of the diagnostic criteria for mental disorders in elite athletes. In consideration of the biopsychosocial context in the etiology, diagnosis, treatment, and prevention of disorders, the model of maladjustment [54] reconceptualized the overtrained state of athletes as an adjustment disorder and, thus, presented a rationale that it should be treated from the perspective of this diagnosis. In contrast, longitudinal cohort research with high-level athletes found a subgroup that were *at risk* (subsyndromal) for adjustment disorder because of a range of stressors (injury, relocation, being on tour and long periods away from home, and the COVID-19 lockdown impact on training and competition; Simons et al, unpublished data, 2020). Therefore, the scope for targeting subsyndromal conditions and psychological symptoms and disorders in athletes can be altered by various measures, diagnostic criteria, and interpretations.

### Psychological Risk, Protective Factors, and Outcome Measures for Tracking

As an example of formative research for tracking, a qualitative study of current and retired elite athletes’ mental health [29] held semistructured in-depth interviews with discussion around their perceptions and expectations of their self, with a focus on achievements and challenges, stressors inside and outside of sporting careers, injuries, transition to life after sport, psychological distress, suicidal ideation, perfectionism, life satisfaction, support, coping strategies, and substance use. Outcome measures for quantitative studies with athletes include knowledge of mental health (ie, disorder and symptom recognition) or behavior regarding mental health (intended or actual help-seeking), mental health competencies (eg, mindfulness and coping) or specific mental health (eg, anxiety, depressive symptoms, and positive affect), and well-being (eg, subjective and psychological well-being domains, and life satisfaction) [41].

Future research is required to conceptualize and gain a better understanding of the combination of multiple stressors and coping resources in athletes. It is also important to verify which factors aid and impede positive adjustment to stressors from a holistic perspective. However, it should not be assumed that there is a need for increased monitoring, surveillance, and medication to enact control (at a cost to recovery). There is a need to promote a balance with a risk assessment and management framework so that the subjective experiences of people with mental health problems are at the forefront of prevention and intervention [55].

### Sporting Culture and Athletic Identity

Culture provides socially constructed myths about natural phenomena, resulting in reshaped systems of belief, which are digested to become part of a worldview and thus influences interpretation of natural phenomena [56]. It is necessary to observe subcultures in athletes to effectively address help-seeking barriers and stigma [42]. For example, athletes *play for each other* in team sports and senior athletes take a leadership role, in addition to coaches and staff, to materialize successful performance. The interconnectedness of sickness and health, and the global effects of COVID-19 means that athletes need each other to adhere to protocols to effectively participate in sports. They also require support and a platform to build a winning culture. However, the “sporting bubble” may distort athletes’ perception of the world (including that mental ill-health is a sign of weakness), which affects their ability to live in it. Therefore, it is important for researchers to gradually peel back the layers acknowledging this group’s attributes such as mental toughness and resilience. Elite athletes may not admit up front to their vulnerabilities such as the effect of the accumulation of pressure and psychological, emotional, professional, and physical stress. Partnered with injuries and recovery, life changes, selection worries, or retirement, their depth of physical and mental strength may be tested such that a breaking point is reached.

Athletes who possess a strong and exclusive level of athletic identity take longer to adapt to their postsport life and are more prone to experiencing psychosocial and career identity difficulties later in life [57]. Athletes who face an involuntary retirement continue to be at the greatest risk of experiencing adjustment issues [58]. The transition out of sport can be a challenging time for athletes [59,60], so preparations for such time should be made well in advance in conjunction with an *exit health examination* [4]. The instability may be inhibited further if they are not in touch with their own feelings and thoughts or these are rendered unacceptable by parents, coaches and staff, peers, or former athletes. The digital age and generational differences also call for innovative ways of connecting athletes with their networks and traditions. Although, this may not always be positive, as high achievers tend to overlay their predisposition to succeed in other areas of their life such that, when the “sporting bubble” has burst, negative coping strategies may be applied with similar intensity (eg, substance misuse).

### Digital Mental Health Solutions

Although not a panacea to mental ill-health issues in athletes or general populations, digital technology has garnered increasing interest and evidence for mental health improvement, especially with younger people [61]. Bevan Jones et al [61] cited a lack of literature to inform the co-design of digital mental health technologies. However, it was established that the involvement of stakeholders is important throughout the life and research cycle of the program. The biggest challenges are the changing face of technology, methods of engaging with diversity in the user group, and evaluation of the co-design process and its impact on the technology.

Risk assessment should consider the dichotomy of digital technology use. It can aid with information and support for those with mental health problems, but it can also be of detriment because of internet-related risk behavior (digital risk) such as bullying or prosuicide websites [62]. Therefore, Aref-Adib et al [62] suggested training and standardized risk pro forma to promote best practice digital risk awareness in mental health care general risk assessments. Digital mental health projects may benefit from a risk management approach that focuses on collaboration, ideation, and innovation, and deals with uncertainty with augmented responses in obtaining objectives, monitoring along the way, communicating, and better integrating and reporting.

Ecological perspectives are important to gather insight for educational strategies to increase mental health literacy [1]. The ecological design perspective provides a conceptual framework within which to investigate more complex interactions between persons and environments [63]. As new ways of being connected to each other and artificial intelligence are becoming more possible and needed, it is important to consider the central setting that digital technology already has in the broader institutional and community contexts. The digital infrastructure is expanding along with its own influence.

The benefits of digital mental health include broad accessibility and choices with free or low-cost availability. It may also be an introduction to psychological intervention that helps participants to overcome stigma or embarrassment. Before technology and changing demand enabled digital mental health to grow, the health industry was dealing with a range of other issues such as inequitable outcomes; unaffordable growth in expenditure; and varied outcomes in wellness, prevention, and intervention. Although there are other pertinent issues, it is important to future proof with data science (programming languages, visualization tools, relevant data platforms). There is also a requirement for practical skills and the knowledge and ability to create complex ecosystems of smart devices as well as the use of the data collected by these devices. However, the *design to innovate* agenda needs to draw from lessons learned and themes such as *the struggle to use digital tools* and *how to overcome difficulties in connecting with them*. A different approach is required to better turn data into information, knowledge, and insight. The design ecosystem needs to give attention to the layout of key aspects of an architecturally designed digital landscape including problem definition, strategy, process and solution, and improvements. Advisory knowledge hubs and dashboards should provide solutions and opportunities to upskill and build capability.

Up-to-date, peer-reviewed online algorithms are useful, for example, in differentiating intentional self-injurious thoughts and behavior into suicidal or nonsuicidal categories [49]. A next frontier in technology is designing artificial intelligence to deliver a range of mental health care services according to algorithms. Artificial intelligence–powered solutions currently include predictive analytics, machine learning, natural language processing or generation, voice recognition and response, virtual personal assistants and chatbots, and diagnosis and recommendation engines. Large-scale and rigorous studies with artificial intelligence–delivered mental health care are still in a preliminary stage [64]. However, Fiske et al [64] noted more evidence is required with regard to patient acceptance and contingent treatment outcomes of embodied artificial intelligence applications in mental health. The social and ethical implications of artificial intelligence remain pertinent issues. Yet, the demand and toll of COVID-19 upon health care workers [50] and the inevitability of further epidemics or disruptions to life calls for virtual mental health care in a practical complementary approach.

Virtual reality (VR) was found to outperform Skype as a therapeutic tool in remote therapy in terms of perceived realism of the session and the degree of presence (face-to-face but in a virtual environment) [65]. The VR concept will be adapted to pioneer digital mental health solutions, including artificial intelligence and deep learning algorithms [66-69], offering promise as a supplementary approach to boosting mental skills and improving the diagnosis and treatment of patients with mental health conditions. A pilot study found VR may benefit the therapeutic alliance [70], especially for people with a psychotic disorder with regard to emotion perception [71]. It was noted that there are limited studies in this field thus far.

There are various mental health apps with links to digital interventions (eg, cognitive behavior therapy and mindfulness exercises). The potential for smartphones to become a real-time diagnostic tool is not yet realized, although the emerging 5G mobile network technology will enable better connection of devices, which will aid this. In the future, 6G may enable the creation of virtual figures or replicas from application of immersive extended reality (XR), high-fidelity mobile holograms, and digital replicas. These HCIs may be programmed for comprehension of algorithms for mental health issues including the intricacies of psychology and psychiatry, somatization, cognition, mind-body connection, behavior, attributes, drive, motivation, attitude, mood, socialization, and personality. However, this is not an exhaustive list. It will be necessary to define and describe HCI and how it impacts user experience design for XR.

Digital solutions with machine learning and artificial intelligence at their core have been developed as flexible tools for identifying patterns in data (via deep learning algorithms) with powerful diagnostic and treatment capabilities to serve large populations at reasonable costs and without human prejudice [72]. The how and why of artificial intelligence delivering virtual mental health care requires an understanding of the Internet of Things (IoT)—the connection of machines for information exchange without human intervention.

The self-improvement capabilities of machine learning and artificial intelligence as new data becomes available is an advantage that assists in early case detection and tracking. However, there is potential for harm and threats arising from the increasing adoption and power [73]. It was recommended by these authors for information literacy and community building in deep learning as tools for getting ahead of deep fakes. To give contexts to the risks, Caldwell et al [73] referred to useful systematic reviews [74], overview of strategic policy in the next 5 years [75], and the cybersecurity risks associated with the interconnectedness of everyday devices (IoT) [76]. A user-friendly description of the technological background of artificial intelligence was presented to instill an understanding of the associated criminal potential and with the intention of reducing potential harm from it [73]. To understand the issue of deep fakes, these authors defined artificial intelligence, machine learning, supervised and unsupervised learning, training data, deep learning, reinforcement learning, active learning, natural language processing, adversarial perturbation, and criminal potential. Patient privacy and the potential for machine learning discrimination requires effective measures to be in place. An emerging example is federated learning whereby an algorithm is trained to function across multiple decentralized devices or servers holding local data samples without exchanging them.

There is an opportunity to innovate developing technologies with the delivery of consistent and cybersecure screening, tracking, and well-being programs in an engaging format that helps users to overcome stigmatization as well as increase mental health literacy, screening honesty, and help-seeking behaviors. In effect, the process of measuring and tracking population mental health is working toward relieving the burden of mental health care “by facilitating the development of relevant and effective interventions and policies before symptoms escalate to clinical levels” as well as “manage and improve the lives and well-being of all people, and not just those with a clinical disorder” [24]. As researchers reimagine scientific or health processes, digital enablement offers promise as a collaborative process that can quickly adapt from client and consumer touchpoint reviews and lessons learned to realizing the total value of its ownership. To grow and evolve a digital mental health ecosystem, it is important to document, store, and publish successful innovation activities; seek ways to increase the rate of adoption; and help shape further opportunities and improvements.

### Recommendations

The following novel recommendations for the design and building of a digital mental health platform and associated screening and tracking technologies and services are intended as a guide for consultation and a consideration for construction. A standard of care and fitness of purpose should be considered with expert and legal advice to ensure a prototype is factual, experienced, and applicable to industry standards before entering a design or construction contract.

#### Athlete-Specific Mental Health Screening Questionnaire

A worldwide-relevant and smartphone compatible athlete-specific mental health screening questionnaire based on the APSQ [30] is recommended to be deployed (weekly), partnered with real-time automation and machine learning models. It should be presented as part of a screening process associated with a well-being framework with consideration of stress and adjustments. A complementary follow-up involving a tracking phase should be implemented weekly with the identified *at-risk* and vulnerable subgroups. It should be designed to improve athlete buy-in with honest feedback and self-evaluation. Referrals to licensed professionals are recommended where clinical levels of psychological distress are identified. The athlete is then to be treated as a patient depending on the symptoms and appropriate treatment recommended by algorithms, such as cognitive behavior therapy or assistance with coping and resilience skills.

The APSQ should be applied as an education concept for the development and validation of subpopulation-specific screening tools. In seeking a consistent and consolidated knowledge management strategy, a hybrid model of care is recommended for the prevention and intervention of stress and adjustment issues as well as psychological disorders. The promotion of mental health improvement goals should be recognized as an investment for athlete well-being programs in addressing issues around engagement, identity, privacy, and confidentiality.

#### Championing of Mental Health Awareness and Antistigma Interventions

Concise guidelines, consensus statements, and brief antistigma interventions are recommended for consolidation and consistency in mental health awareness. It is recommended to highlight the range of mental health states with elite athlete and mental health champions to raise mental health awareness in society. It is recommended to specifically target peculiar subgroups of athletes with more focused prevention and tailored interventions that recognize varied career stages and mental health states.

#### Pivot From Existing Subpopulation Research to Quickly Respond to Disruptive Global Events

Further research is recommended to expand the finding that high-level athletes are at increased vulnerability to mental ill-health, yet there are positive adjustments and resilience in response to *overwhelming stress*, resulting in a subsyndromal condition (Simons et al, unpublished data, 2020). It is recommended to pivot from existing studies such as with college student, high-level, and elite athletes to investigate and articulate “how behaviors and mental health change and interact in the face of monumental adverse global events” [19]. Verification of behavior research is required such as a study with the Circadian Rhythm for Mood app, which found that a wearable device is effective in the prevention, intervention, and improvement of mood disorders [77].

In targeting *at-risk* subpopulations such as athletes for early intervention, it is recommended to implement brief, valid, and reliable smartphone-compatible online surveys (with consideration of the offline options such as paper and text messaging alternatives for the underserved). It should be tailored to the relevant psychological risk and protective factors. Tracking (monitoring of symptoms, in particular where there is an indication of high stress and adjustment issues but not psychological distress or disorder) is recommended to be run by unlicensed professionals such as academic researchers or from other areas of psychology, psychiatry, or mental health that do not require direct clinical contact with patients. A rationale for tracking is that descriptive and experimental research is required to increase positive engagement with *at-risk* and vulnerable subpopulations, support mental health care services, and counter the psychological impact of disruptive global events such as the COVID-19 pandemic.

#### Integration of Subpopulation and General Population Research With Algorithms

Valid and reliable subpopulation research should be compared with findings from the general population. Blind randomized longitudinal studies with large-scale data collection via MHQ screening in general populations may expand its validation. Evidence- or consensus-based guidelines for diagnosis and management of mental health symptoms and disorders are recommended to evolve into standardized, up-to-date online algorithms. Real-time automation with machine learning models is recommended to apply the MHQ to test its effectiveness in tracking compared with results from clinical psychologists and psychiatrists as well as unlicensed professionals, who may contribute more of a supporting role. The intent is to inform effective and efficient pathways of mental health care with valid, reliable, and replicable identification of at-risk individuals and subgroups, and assess the impact of any support and wellness programs.

The MHQ provides for the geographical, cultural, and experiential factors of the general population, and covers the complete range of clinical mental health symptoms as well as positive mental assets [24]. It is recommended for general population self-screening because it encompasses a fluid state of how these symptoms and assets interact based on an extensive review of the way mental health is assessed in clinical and research fields [78]. The MHQ is an epidemiological mental health assessment tool that provides personalized insight into an individual’s mental health partnered with feedback that is generated based on the individual scoring profile. The delivery of solutions for influencing positive mental health is recommended to consider the mixed linear model of smartphone mobile sensing and self-reported mental health questions [19], and a hybrid model of care [10,20-22] as part of a general guide.

#### Development and Evaluation of Digital Mental Health Platforms

A road map [25] and integrated blueprint [26] for platform-based solutions are urgently needed to strengthen mental health systems with consolidation, consensus, and concise guidelines. There are opportunities for growth in digital mental health risk surveillance (screening and tracking) extending from an honest and transparent platform, which promotes trust and confidentiality. It is recommended to progress opportunities in digital mental health (especially with platforms) while acknowledging that there are constraints and rigorous evaluation is required [26]. It is recognized that these emerging developments are “not explicitly covered by the existing guidelines” [21]. However, it is recommended that a certification framework, applicable industry standards, and expert advice (eg, a road map, integrated blueprint, and innovation ecosystem for digital mental health services) guide entrepreneurial activities. In the absence of a government-endorsed certification framework, it is recommended to consult with the terms and conditions of a reliable example that has been evaluated to an international standard. It is also important to ensure there is a synergy of activities for mental health care with efforts in outreach and dissemination of peer-reviewed literature as well as development of a skilled, digitally capable workforce.

A new type of patient-consumer is fueling remote screening and tracking’s rapid growth. Although there is a wide range of choices available for digital mental health, there is an absence of a useful and reliable athlete-specific digital mental health platform. If a prototype is developed, it should serve as an example to other subpopulations for tailored prevention and intervention. Mental health resources and services should be sourced from trusted organizations, including links to evidence-based apps and programs, forums, phone chats, emails, and websites.

Digital mental health requires consultative leadership in advising, designing, and delivering the platforms and infrastructure for a transformation in client and consumer delivery. An organization’s track record is important for building or adopting a brand for an individual. It is recommended to not be reliant on a small, distant vendor for a core platform, which could be easily disrupted and cause a negative flow-on effect. Therefore, a well-established company may help build trust in the adoption of digital mental health technologies. A future-ready technology and health company is recommended for an end-to-end service, offering and drawing on depth and capability to deliver on promises.

#### Web-Based and Smartphone Mental Health App Considerations

Web-based and smartphone mental health apps are recommended to be presented in a visually engaging way with an appropriate balance of human characteristics in the graphics (representative of diversity and ethnicity). Brief scalable survey questions, which can be skipped or with options to go to the next or previous one, may be supported with an open-ended answer box that prompts the user to explain their thoughts and feelings after a score is provided. An information package should include the conditions and terms of engagement, and links to the app, app help, translated information, resources, what it is for, how it works, frequently asked questions (FAQs), deleting the app, and privacy. There should also be information provided about data collection, processing, and storage, with details on the purpose, data source and access, storage period and location, contact information, and complaints.

#### Human-Computer Interactions in a Hybrid Model of Care

The digital therapeutic alliance (DTA) as a concept [79-83] suggests that there is a future for HCI, but there is a need for testing of emerging interactive techniques. More research is required to support acceptability, feasibility, and safety around digital interventions [84]. The efficacy of a hybrid model of care requires evaluation of its impact on the therapeutic alliance, clinical and social outcomes, cost-effectiveness, and engagement [23]. A therapeutic alliance may be refined in digital interventions with its own unique features (although further investigation is required to affirm this) [69].

Some mental health functional specialists may be required to lead, upskill, and strategize in data science and artificial intelligence. It is important to understand the potential and limitations of smart devices and apply a design-thinking approach as a way to come up with creative solutions. The design, implementation, and analysis of smart systems that gather data can inform and drive more effective and insightful mental health policies and strategies, for example, Lederman et al’s [85] suggestions to aim for adaptability of multifaceted tools through *concordance in HCI*. This concept takes account of the self-determination theory and is useful for design improvements from peer and moderator support as well as automated feedback [85]. Future developments with digital interventions are recommended to measure HCI for engagement and adherence effectiveness as well as individual client and consumer satisfaction with the DTA experience, including feedback on its functionality, trustworthiness, and expertise as well as confidence in its recommendations.

It is recommended for longitudinal research of screening and tracking with real-time automation and machine learning to determine whether there is improvement in engagement levels, ongoing participation rates, mental health literacy, and help-seeking behaviors. This strategy is recommended to be tailored toward a hybrid model of care including self-help, face-to-face, and digital interventions with *at-risk* and vulnerable subpopulations.

#### Artificial Intelligence, Deep Learning Algorithms, VR, and Digital Interventions

Artificial intelligence, object-oriented, and information systems as well as deep learning algorithms are increasingly of interest for therapeutic outcomes from digital interventions. It is recommended for an experimental rationale to better understand the *bond* within the human-smartphone connection. The IoT structure is recommended for database, statistical, and communication systems, including edge devices, data mining, and visualization as well as security and cloud computing.

The ecological perspective is suitable to be adopted with the co-design of digital mental health technologies to describe how the system or product works within its external environment. For example, VR modules are used for the treatment of multiple conditions from addiction to eating disorders, depression and anxiety, stress, and trauma as well as for immersion and repetition of scenes that may be useful for sport performance and mindfulness. It is recommended to expand research with VR and emerging technologies to produce specialized immersive interactive experiences for *at-risk* and vulnerable subpopulations, and to quantify the effect of creative problem solving and critical reflection upon stress and adjustment to see if there is a relationship with subsyndromal conditions or symptoms and disorders.

In determining a DTA for virtual clinics, it is recommended to consult up-to-date algorithms especially for evidence-based psychological protective and risk factors. A subset of HCI, affective computing, is recommended to be included in the co-design for user state–tailored response and an appealing balance of artificial intelligence and human characteristics such as traits, emotions, and intentions.

### Conclusions

The novel finding that the COVID-19 pandemic had a dichotomous impact on high-level athletes’ mental well-being, with a period of *overwhelming stress* followed by a return to baseline well-being during lockdown (Simons et al, unpublished data, 2020), suggests a peculiarity in an *at-risk* but resilient subgroup of a subpopulation who are resourceful in achieving positive adjustments. A next step is validation with elite athletes. Brief psychological strain screening tools such as the APSQ should extend with stress and adjustment-focused methods [17,18]. The unrealized potential of this early intervention strategy should also be more generally explored (eg, MHQ) for how it may assist the management of mental health (from mental ill-health to positive functioning).

Future research should draw comparisons between athletes and the general population with regard to broadening the scope of mental health, especially symptom and disorder interpretation. There is a high potential for reliable and evaluated digital mental health platforms to inform associated prevention and early intervention strategies. Collaboration between humans and machines will be critical to the innovation of mental health care in the future. Abilities or willingness to embrace and adopt machine learning (with real-time automation) and artificial intelligence in a hybrid model of care should be guided by the right relevance in a road map and integrated blueprint to drive valid and reliable studies and address concerns over the potential for threats or harm. Studies adopting inclusive design or human-centric design principles should demonstrate how embracing digital mental health (especially deriving from digital platforms and algorithms from machine learning and artificial intelligence) is a critical success factor in a post–COVID-19 world.

Sports have a great potential to become more than a fertile platform for awareness of mental health issues and education for seeking help. Athletes are a suitable subpopulation for which to implement digital mental health solutions for stress and adjustment as a complement to prevention and intervention. Validation of the dichotomy of athlete mental health problems and resilience (in or outside of a pandemic-affected situation) offers a dual opportunity to advance digital mental health screening and tracking, and enact faster and better treatment algorithms for *at-risk* and vulnerable subpopulations.

## Abbreviations

AIS: Australian Institute of Sport
APSQ: Athlete Psychological Strain Questionnaire
DSM: Diagnostic and Statistical Manual of Mental Disorders
DTA: digital therapeutic alliance
FAQ: frequently asked question
HCI: human-computer interaction
ICD: International Classification of Diseases
IoT: Internet of Things
MHQ: Mental Health Quotient
PTSD: posttraumatic stress disorder
VR: virtual reality
XR: extended reality
